# A systematic literature review on the impact of AI models on the security of code generation

**DOI:** 10.3389/fdata.2024.1386720

**Published:** 2024-05-13

**Authors:** Claudia Negri-Ribalta, Rémi Geraud-Stewart, Anastasia Sergeeva, Gabriele Lenzini

**Affiliations:** ^1^Security and Trust, University of Luxembourg, Luxembourg, Luxembourg; ^2^École Normale Supérieure, Paris, France; ^3^Faculty of Humanities, Education, and Social Sciences, University of Luxembourg, Luxembourg, Luxembourg

**Keywords:** artificial intelligence, security, software engineering, programming, code generation

## Abstract

**Introduction:**

Artificial Intelligence (AI) is increasingly used as a helper to develop computing programs. While it can boost software development and improve coding proficiency, this practice offers no guarantee of security. On the contrary, recent research shows that some AI models produce software with vulnerabilities. This situation leads to the question: How serious and widespread are the security flaws in code generated using AI models?

**Methods:**

Through a systematic literature review, this work reviews the state of the art on how AI models impact software security. It systematizes the knowledge about the risks of using AI in coding security-critical software.

**Results:**

It reviews what security flaws of well-known vulnerabilities (e.g., the MITRE CWE Top 25 Most Dangerous Software Weaknesses) are commonly hidden in AI-generated code. It also reviews works that discuss how vulnerabilities in AI-generated code can be exploited to compromise security and lists the attempts to improve the security of such AI-generated code.

**Discussion:**

Overall, this work provides a comprehensive and systematic overview of the impact of AI in secure coding. This topic has sparked interest and concern within the software security engineering community. It highlights the importance of setting up security measures and processes, such as code verification, and that such practices could be customized for AI-aided code production.

## 1 Introduction

Despite initial concerns, increasingly, many organizations rely on artificial intelligence (AI) to enhance the operational workflows in their software development life cycle and to support writing software artifacts. One of the most well-known tools is GitHub Copilot. It is created by Microsoft relies on OpenAI's Codex model, and is trained on open-source code publicly available on GitHub (Chen et al., 2021). Like many similar tools—such as CodeParrot, PolyCoder, StarCoder—Copilot is built atop a large language model (LLM) that has been trained on programming languages. Using LLMs for such tasks is an idea that dates back at least as far back as the public release of OpenAI's ChatGPT.

However, using automation and AI in software development is a double-edged sword. While it can improve code proficiency, the quality of AI-generated code is problematic. Some models introduce well-known vulnerabilities, such as those documented in MITRE's Common Weakness Enumeration (CWE) list of the top 25 “most dangerous software weaknesses.” Others generate so-called “stupid bugs,” naïve single-line mistakes that developers would qualify as “stupid” upon review (Karampatsis and Sutton, 2020).

This behavior was identified early on and is supported to a varying degree by academic research. Pearce et al. (2022) concluded that 40% of the code suggested by Copilot had vulnerabilities. Yet research also shows that users trust AI-generator code more than their own (Perry et al., 2023). These situations imply that new processes, mitigation strategies, and methodologies should be implemented to reduce or control the risks associated with the participation of generative AI in the software development life cycle.

It is, however, difficult to clearly attribute the blame, as the tooling landscape evolves, different training strategies and prompt engineering are used to alter LLMs behavior, and there is conflicting if anecdotal, evidence that human-generated code could be just as bad as AI-generated code.

This systematic literature review (SLR) aims to critically examine how the code generated by AI models impacts software and system security. Following the categorization of the research questions provided by Kitchenham and Charters (2007) on SLR questions, this work has a 2-fold objective: analyzing the impact and systematizing the knowledge produced so far. Our main question is:

“**How does the code generation from AI models impact the cybersecurity of the software process?**”

This paper discusses the risks and reviews the current state-of-the-art research on this still actively-researched question.

Our analysis shows specific trends and gaps in the literature. Overall, there is a high-level agreement that **AI models do not produce safe code** and **do introduce vulnerabilities**, despite mitigations. Particular vulnerabilities appear more frequently and prove to be more problematic than others (Pearce et al., 2022; He and Vechev, 2023). Some domains (e.g., hardware design) seem more at risk than others, and there is clearly an imbalance in the efforts deployed to address these risks.

This work stresses the importance of relying on dedicated security measures in current software production processes to mitigate the risks introduced by AI-generated code and highlights the limitations of AI-based tools to perform this mitigation themselves.

The article is divided as follows: we first introduce the reader to AI models and code generation in Section 2 to proceed to explain our research method in Section 3. We then present our results in Section 4. In Section 5 we discuss the results, taking in consideration AI models, exploits, programming languages, mitigation strategies and future research. We close the paper by addressing threats to validity in Section 6 and conclusion in Section 7.

## 2 Background and previous work

### 2.1 AI models

The sub-branch of AI models that is relevant to our discussion are *generative* models, especially large-language models (LLMs) that developed out of the attention-based transformer architecture (Vaswani et al., 2017), made widely known and available through pre-trained models (such as OpenAI's GPT series and Codex, Google's PaLM, Meta's LLaMA, or Mistral's Mixtral).

In a transformer architecture, inputs (e.g., text) are converted to tokens^1^ which are then mapped to an abstract latent space, a process known as *encoding* (Vaswani et al., 2017). Mapping back from the latent space to tokens is accordingly called *decoding*, and the model's parameters are adjusted so that encoding and decoding work properly. This is achieved by feeding the model with human-generated input, from which it can learn latent space representations that match the input's distribution and identify correlations between tokens.

Pre-training amortizes the cost of training, which has become prohibitive for LLMs. It consists in determining a reasonable set of weights for the model, usually through autocompletion tasks, either autoregressive (ChatGPT) or masked (BERT) for natural language, during which the model is faced with an incomplete input and must correctly predict the missing parts or the next token. This training happens once, is based on public corpora, and results in an initial set of weights that serves as a baseline (Tan et al., 2018). Most “open-source” models today follow this approach.^2^

It is possible to fine-tune parameters to handle specific tasks from a pre-trained model, assuming they remain within a small perimeter of what the model was trained to do. This final training often requires human feedback and correction (Tan et al., 2018).

The output of a decoder is not directly tokens, however, but a probability distribution over tokens. The *temperature* hyperparameter of LLMs controls how much the likelihood of less probable tokens is amplified: a high temperature would allow less probable tokens to be selected more often, resulting in a less predictable output. This is often combined with nucleus sampling (Holtzman et al., 2020), i.e., requiring that the total sum of token probabilities is large enough and various penalty mechanisms to avoid repetition.

Finally, before being presented to the user, an output may undergo one or several rounds of (possibly non-LLM) filtering, including for instance the detection of foul language.

### 2.2 Code generation with AI models

With the rise of generative AI, there has also been a rise in the development of AI models for code generation. Multiple examples exist, such as Codex, Polycoder, CodeGen, CodeBERT, and StarCoder, to name a few (337, Xu, Li). These new tools should help developers of different domains be more efficient when writing code—or at least expected to (Chen et al., 2021).

The use of LLMs for code generation is a domain-specific application of generative methods that greatly benefit from the narrower context. Contrary to natural language, programming languages follow a well-defined syntax using a reduced set of keywords, and multiple clues can be gathered (e.g., filenames, other parts of a code base) to help nudging the LLM in the right direction. Furthermore, so-called boilerplate code is not project-specific and can be readily reused across different code bases with minor adaptations, meaning that LLM-powered code assistants can already go a long way simply by providing commonly-used code snippets at the right time.

By design, LLMs generate code based on their training set (Chen et al., 2021).^3^ In doing so, there is a risk that sensitive, incorrect, or dangerous code is uncritically copied verbatim from the training set or that the “minor adaptations” necessary to transfer code from one project to another introduces mistakes (Chen et al., 2021; Pearce et al., 2022; Niu et al., 2023). Therefore, generated code may include security issues, such as well-documented bugs, malpractices, or legacy issues found in the training data. A parallel issue often brought up is the copyright status of works produced by such tools, a still-open problem that is not the topic of this paper.

Similarly, other challenges and concerns have been highlighted by different academic research. From an educational point of view, some concerns are that using AI code generation models may impact acquiring bad security habits between novice programmers or students (Becker et al., 2023). However, the usage of such models can also help lower the entry barrier to the field (Becker et al., 2023). Similarly, cite337 has suggested that using AI code generation models does not output secure code all the time, as they are non-deterministic, and future research on mitigation is required (Pearce et al., 2022). For example, Pearce et al. (2022) was one of the first to research this subject.

There are further claims that it may be possible to use by cyber criminal (Chen et al., 2021; Natella et al., 2024). In popular communication mediums, there are affirmations that ChatGPT and other LLMs will be “useful” for criminal activities, for example Burgess (2023). However, these tools can be used defensively in cyber security, as in ethical hacking (Chen et al., 2021; Natella et al., 2024).

## 3 Research method

This research aims to systematically gather and analyze publications that answer our main question: “**How does the code generation of AI models impact the cybersecurity of the software process?**” Following Kitchenham and Charters (2007) classification of questions for SLR, our research falls into the type of questions of “Identifying the impact of technologies” on security, and “Identifying cost and risk factors associated with a technology” in security too.

To carry out this research, we have followed different SLR guidelines, most notably Wieringa et al. (2006), Kitchenham and Charters (2007), Wohlin (2014), and Petersen et al. (2015). Each of these guidelines was used for different elements of the research. We list out in a high-level approach which guidelines were used for each element, which we further discuss in different subsections of this article.

- For the general structure and guideline on how to carry out the SLR, we used Kitchenham and Charters (2007). This included exclusion and inclusion criteria, explained in Section 3.2 ;
- The identification of the Population, Intervention, Comparison, and Outcome (PICO) is based both in Kitchenham and Charters (2007) and Petersen et al. (2015), as a framework to create our search string. We present and discuss this framework in Section 3.1 ;
- The questions and quality check of the sample, we used the research done by Kitchenham et al. (2010), which we describe in further details at Section 3.4 ;
- The taxonomy of type of research is from Wieringa et al. (2006) as a strategy to identify if a paper falls under our exclusion criteria. We present and discuss this taxonomy in Section 3.2. Although their taxonomy focuses on requirements engineering, it is broad enough to be used in other areas as recognized by Wohlin et al. (2013);
- For the snowballing technique, we used the method presented in Wohlin (2014), which we discuss in Section 3.3 ;
- Mitigation strategies from Wohlin et al. (2013) are used, aiming to increase the reliability and validity of this study. We further analyze the threats to validity of our research in Section 6.

In the following subsections, we explain our approach to the SLR in more detail. The results are presented in Section 4.

### 3.1 Search planning and string

To answer our question systematically, we need to create a search string that reflects the critical elements of our questions. To achieve this, we thus need to frame the question in a way that allows us to (1) identify keywords, (2) identify synonyms, (3) define exclusion and inclusion criteria, and (4) answer the research question. One common strategy is the PICO (population, intervention, comparison, outcome) approach (Petersen et al., 2015). Originally from medical sciences, it has been adapted for computer science and software engineering (Kitchenham and Charters, 2007; Petersen et al., 2015).

To frame our work with the PICO approach, we follow the methodologies outlined in Kitchenham and Charters (2007) and Petersen et al. (2015). We can identify the set of keywords and their synonyms by identifying these four elements, which are explained in detail in the following bullet point.

- Population: Cybersecurity.
- Following Kitchenham and Charters (2007), a population can be an area or domain of technology. Population can be very specific.
- Intervention: AI models.
- Following Kitchenham and Charters (2007) “The intervention is the software methodology/tool/technology, such as the requirement elicitation technique.”
- Comparison: we compare the security issues identified by the code generated in the research articles. In Kitchenham and Charters (2007) word, “This is the software engineering methodology/tool/technology/procedure with which the intervention is being compared. When the comparison technology is the conventional or commonly-used technology, it is often referred to as the ‘control' treatment.”
- Outcomes: A systematic list of security issues of using AI models for code generation and possible mitigation strategies.
- Context: Although not mandatory (per Kitchenham and Charters, 2007) in general we consider code generation.

With the PICO elements done, it is possible to determine specific keywords to generate our search string. We have identified three specific sets: security, AI, and code generation. Consequently, we need to include synonyms of these three sets for generating the search string, taking a similar approach as Petersen et al. (2015). The importance of including different synonyms arises from different research papers referring to the same phenomena differently. If synonyms are not included, essential papers may be missed from the final sample. The three groups are explained in more detail:

- Set 1: search elements related to security and insecurity due to our population of interest and comparison.
- Set 2: AI-related elements based on our intervention. This set should include LLMs, generative AI, and other approximations.
- Set 3: the research should focus on code generation.

With these three sets of critical elements that our research focuses on, a search string is created. We constructed the search string by including synonyms based on the three sets (as seen in Table 1). In a concurrent manner, while identifying the synonyms, we create the search string. Through different iterations, we aim at achieving the “golden” string, following a test-retest approach by Kitchenham et al. (2010). In every iteration, we checked if the vital papers of our study were in the sample. The final string was selected based on the new synonym that would add meaningful results. For example, one of the iterations included “hard*,” which did not add any extra article. Hence, it was excluded. Due to space constraints, the different iterations are available in the public repository of this research. The final string, with the unique query per database, is presented in Table 2.

**Table 1:** Keywords and synonyms.

**Keyword**	**Synonyms**
Artificial intelligence	AI, large language models, LLM, and LLMS
Code generation	Code creation, generate code, code production, code writing, and code quality
Cyber security	Secure, insecure, security, vulnerab*, threat*, exploit, fault*, and failure

^*^ denotes the wildcard in the search.

**Table 2:** Search string per database.

**Database**	**Search string**
IEEE Xplore	(“Abstract”:LLM OR AI OR “artificial intelligence” OR LLMs OR “large language models”)AND(“Abstract”:“code generation” OR “code creation” OR “generate code” OR “code writing” OR “code production” OR “code correction” OR “code quality”)AND(“Abstract”:security OR “cyber security” OR insecure OR secure OR insecurity OR vulnerab* OR threat* OR exploit OR fault OR failure)
ACM	[[Abstract: “ai”] OR [Abstract: “large language models”] OR [Abstract: “llm”] OR [Abstract: “artificial intelligence”]] AND [[Abstract: “code generation”] OR [Abstract: “code creation”] OR [Abstract: “generate code”] OR [Abstract: “code production”] OR [Abstract: “code correction”] OR [Abstract: “code quality”]] AND [[Abstract: “security”] OR [Abstract: “cyber security”] OR [Abstract: “secure”] OR [Abstract: “insecure”] OR [Abstract: vulnerab*] OR [Abstract: threat*] OR [Abstract: exploit] OR [Abstract: fault*] OR [Abstract: failure]]
SCOPUS	(TITLE-ABS-KEY(“LLM”) OR TITLE-ABS-KEY(“artificial intelligence”) OR TITLE-ABS-KEY(“large language models”) OR TITLE-ABS-KEY(“LLMs”)) AND (TITLE-ABS-KEY(“code generation”) OR TITLE-ABS-KEY(“code creation”) OR TITLE-ABS-KEY(“generate code”) OR TITLE-ABS-KEY(“code writing”) OR TITLE-ABS-KEY(“code quality”) OR TITLE-ABS-KEY(“code correction”) OR TITLE-ABS-KEY(“code production”)) AND (TITLE-ABS-KEY(“security”) OR TITLE-ABS-KEY(“cyber security”) OR TITLE-ABS-KEY(“security”) OR TITLE-ABS-KEY(“insecure”) OR TITLE-ABS-KEY(vulnerab*) OR TITLE-ABS-KEY(exploit*) OR TITLE-ABS-KEY(fault*) OR TITLE-ABS-KEY(“failure”))

For this research, we selected the following databases to gather our sample: IEEE Explore, ACM, and Scopus (which includes Springer and ScienceDirect). The databases were selected based on their relevance for computer science research, publication of peer-reviewed research, and alignment with this research objective. Although other databases from other domains could have been selected, the ones selected are notably known in computer science.

### 3.2 Exclusion and inclusion criteria

The exclusion and inclusion criteria were decided to align our research objectives. Our interest in excluding unranked venues is to avoid literature that is not peer-reviewed and act as a first quality check. This decision also applies to gray literature or book chapters. Finally, we excluded opinion and philosophical papers, as they do not carry out primary research. Table 3 shows are inclusion and exclusion criteria.

**Table 3:** Inclusion and exclusion criteria.

**Inclusion**	**Exclusion**
• Studies that are about AI code generation; • Studies that explicitly address security elements as the main object of study; • Papers written in English.	• Study not peer-reviewed, including books and book chapters; • Study not available online; • Studies about AI models in general; • Secondary research (SLR, summaries, and guidelines/templates); • Unranked venues; • Gray literature; • Opinion papers and philosophical papers.

We have excluded articles that address AI models or AI technology in general, as our interest—based on PICO—is on the security issue of AI models in code generation. So although such research is interesting, it does not align with our main objective.

For identifying the secondary research, opinion, and philosophical papers—which are all part of our exclusion criteria in Table 3—we follow the taxonomy provided by Wieringa et al. (2006). Although this classification was written for the requirements engineering domain, it can be generalized to other domains (Wieringa et al., 2006). In addition, apart from helping us identify if a paper falls under our exclusion criteria, this taxonomy also allows us to identify how complete the research might be. The classification is as follows:

- *Solution proposal:* Proposes a solution to a problem (Wieringa et al., 2006). “The solution can be novel or a significant extension of an existing technique (Petersen et al., 2015).”
- *Evaluation research:* “This is the investigation of a problem in RE practice or an implementation of an RE technique in practice [...] novelty of the knowledge claim made by the paper is a relevant criterion, as is the soundness of the research method used (Petersen et al., 2015).”
- *Validation research:* “This paper investigates the properties of a solution proposal that has not yet been implemented... (Wieringa et al., 2006).”
- *Philosophical papers:* “These papers sketch a new way of looking at things, a new conceptual framework (Wieringa et al., 2006).”
- *Experience papers:* Is where the authors publish their experience over a matter. “In these papers, the emphasis is on what and not on why (Wieringa et al., 2006; Petersen et al., 2015).”
- *Opinion papers:* “These papers contain the author's opinion about what is wrong or good about something, how we should do something, etc. (Wieringa et al., 2006).”

### 3.3 Snowballing

Furthermore, to increase the reliability and validity of this research, we applied a forward snowballing technique (Wohlin et al., 2013; Wohlin, 2014). Once the first sample (start set) has passed an exclusion and inclusion criteria based on the title, abstract, and keyword, we forward snowballed the whole start set (Wohlin et al., 2013). That is to say; we checked which papers were citing the papers from our starting set, as suggested by Wohlin (2014). For this section, we used Google Scholar.

In the snowballing phase, we analyzed the title, abstract, and key words of each possible candidate (Wohlin, 2014). In addition, we did an inclusion/exclusion analysis based on the title, abstract, and publication venue. If there was insufficient information, we analyzed the full text to make a decision, following the recommendations by Wohlin (2014).

Our objective with the snowballing is to increase the reliability and validity. Furthermore, some articles found through the snowballing had been accepted at different peer-reviewed venues but had not been published yet in the corresponding database. This is a situation we address at Section 6.

### 3.4 Quality analysis

Once the final sample of papers is collected, we proceed with the quality check, following the procedure of Kitchenham and Charters (2007) and Kitchenham et al. (2010). The objective behind a quality checklist if 2-fold: “to provide still more detailed inclusion/exclusion criteria” and act “as a means of weighting the importance of individual studies when results are being synthesized (Kitchenham and Charters, 2007).” We followed the approach taken by Kitchenham et al. (2010) for the quality check, taking their questions and categorizing. In addition, to further adapt the questionnaire to our objectives, we added one question on security and adapted another one. The questionnaire is properly described at Table 4. Each question was scored, according to the scoring scale defined in Table 5.

**Table 4:** Quality criteria questionnaire.

• (Question on design, data collection, data analysis:) Do the authors describe the research methods?^*^ • Do the authors describe the data collection procedure and define the measurements? (Applicable for validation and evaluation papers)^*^ • Do the authors define the (security) analysis procedure?^†^ • Do the authors discuss threats to validity and limitations?^*^	• (Question on aims:) Do the authors clearly state the aims of the research?^*^ • (Question on study outcomes:) Do the authors state the findings clearly?^*^ • Is the evidence of this research be used by others?^*^ • (Our question:) Does the study explicitly address a cybersecurity concern?^‡^

^*^Are questions obtained from Kitchenham et al. (2010), ^†^are slightly modified questions based from Kitchenham et al. (2010), and ^‡^are original question of this research.

**Table 5:** Quality criteria assessment.

**Score value**	**Description**
*Fully* (1 point)	The article addresses the question and provides enough details for the reader to understand;
*Mostly* (0.66)	The article addresses the most relevant concerns to questions; however, certain details are missing;
*Somewhat* (0.33)	The article addresses some of the concerns to answer the question; however, it leaves out vital concerns;
*No* (0)	The article does not address the question in any detail.

The quality analysis is done by at least two authors of this research, for reliability and validity purposes (Wohlin et al., 2013).

### 3.5 Data extraction

To answer the main question and extract the data, we have subdivided the main question, to answer it. This allows us to extract information and summarize it systematically; we created an extract form in line with (Kitchenham and Charters, 2007; Carrera-Rivera et al., 2022). The data extraction form is presented in Table 6.

**Table 6:** Data extraction form and type of answer.

**Question**	**Type of answer**
**About the AI model**	
Which AI model does it study	String
Only LLms?	Boolean
Which specific LLM	String field
Any extra comments on the AI model section	String field
**Identification of security issues**	
What type of security concern is addressed [General]	String field
How was the concern identified?	String field
Which methodology is used for verifying the identified concern?	String field
**Vulnerabilities identified and mitigation strategies**	
Is it specific for one programming language?	Boolean
Which programming language(s)	Select many fields
What vulnerability(ies) is(are) addressed?	String field
Technical, socio-technical, or human vulnerabilities?	Select many fields
Are they known vulnerability(ies)?	Boolean
If known, which vulnerability(ies)?	String field
Do the authors present a new exploit	Boolean
If it is a new exploit, summarize and describe it	String field
Are mitigation strategies suggested?	Boolean
If mitigation strategies are suggested, please provide details	String field
Are these mitigation strategy for a specific AI model?	Boolean
At what level are the mitigation strategy suggested	Select many fields
**Extra elements**	
Extra comments	String field

The data extraction was done by at least two researchers per article. Afterward, the results are compared, and if there are “disagreements, [they must be] resolved either by consensus among researchers or arbitration by an additional independent researcher (Kitchenham and Charters, 2007).”

## 4 Results

### 4.1 Search results

**Figure 1:**
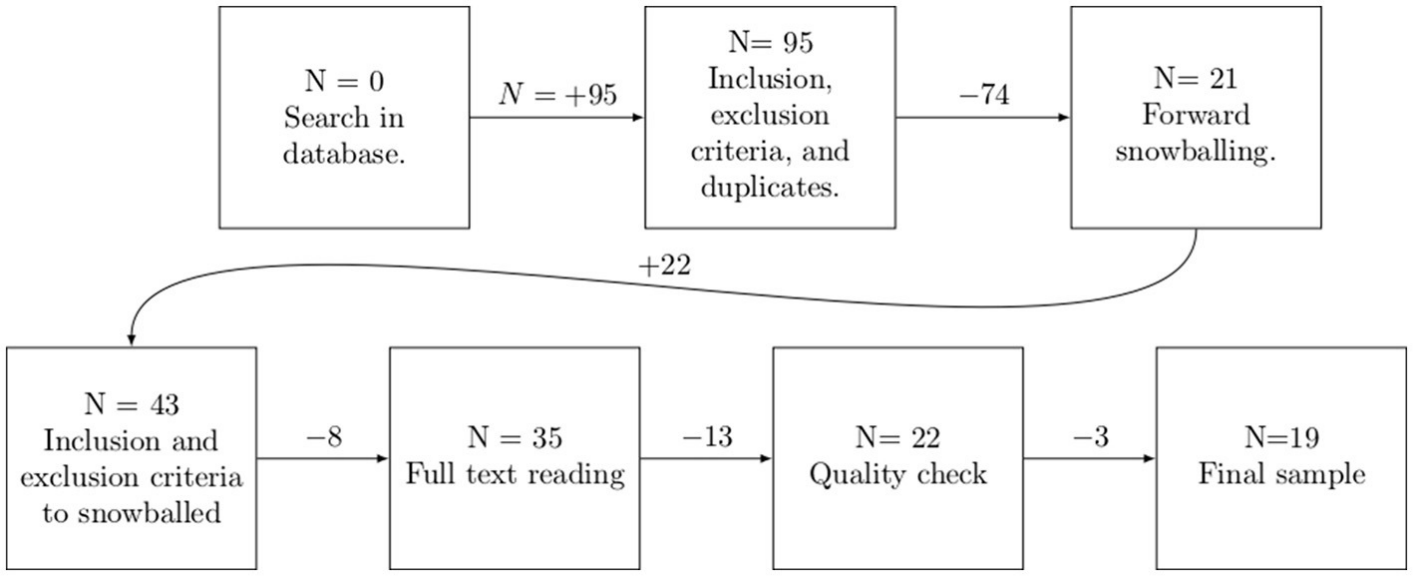
Selection of sample papers for this SLR.

The search and recollection of papers were done during the last week of November 2023. Table 7 shows the total number of articles gathered per database. The selection process for our final samples is exemplified in Figure 1.

**Table 7:** Search results per database.

**Database**	**IEEE**	**ACM**	**SCOPUS**
Search results	49	5	41

The total number of articles in our first round, among all the databases, was 95. We then identified duplicates and applied our inclusion and exclusion criteria for the first round of selected papers. This process left us with a sample of 21 articles.

These first 21 artcles are our starting set, from which we proceeded for a forward snowballing. We snowballed each paper of the starting set by searching Google Scholar to find where it had been cited. The selected papers at this phase were based on the title, abstract, based on Wohlin (2014). From this step, 22 more articles were added to the sample, leaving 43 articles. We then applied inclusion and exclusion criteria to the new snowballed papers, that left us with 35 papers. We discuss this high number of snowballed papers at Section 6.

At this point, we read all the articles to analyze if they should pass to the final phase. In this phase, we discarded 12 articles deemed out of scope for this research, leaving us with 23 articles for quality check. For example, they would not focus on cybersecurity, code generation, or the usage of AI models for code generation.

At this phase, three particular articles (counted among the eight articles previously discarded) sparked discussion between the first and fourth authors regarding whether they were within the scope of this research. We defined AI code generation as artifacts that suggest or produce code. Hence, those artifacts that use AI to check and/or verify code, and vulnerability detection without suggesting new code are not within scope. In addition, the article's main focus should be on code generation and not other areas, such as code verification. So, although an article might discuss code generation, the paper was not accepted as it was not the main topic. As a result, two of the three discussion articles were accepted, and one was rejected.

### 4.2 Quality evaluation

We carried out a quality check for our preliminary sample of papers (*N* = 23) as detailed at Section 3.4. Based on the indicated scoring system, we discarded articles that did not pass 50% of the total possible score (four points). If there were disagreements in the scoring, these were discussed and resolved between authors. Each paper's score details are provided in Table 8, for transparency purposes (Carrera-Rivera et al., 2022). Quality scores guides us on where to place more weight of importance, and on which articles to focus (Kitchenham and Charters, 2007). The final sample is of *N* = 19.

**Table 8:** Quality scores of the final sample.

**References**	**Q1**	**Q2**	**Q3**	**Q4**	**Q5**	**Q6**	**Q7**	**Q8**	**Final score**
Pa Pa et al. (2023)	1	0.66	0.33	1	0.33	0.33	0.66	0	4.31
Siddiq et al. (2022)	1	1	1	0.33	1	1	1	0.33	6.66
Pearce et al. (2022)	1	1	1	1	1	1	1	1	8
Jia et al. (2023)	1	1	1	1	0.66	0	0.66	0	5.32
Jha and Reddy (2023)	1	1	1	1	1	0.33	1	0.33	6.66
Perry et al. (2023)	1	1	1	1	1	0	1	1	7
Storhaug et al. (2023)	1	1	0.66	1	0.33	0.33	0.66	0.33	5.31
Pearce et al. (2023)	1	1	1	1	0.66	1	1	1	7.66
Botacin (2023)	1	1	1	1	1	0.33	1	1	7.33
Tony et al. (2022)	1	1	1	1	1	1	1	1	8
Wu et al. (2023)	1	0.66	1	1	1	0.33	1	0.66	6.65
Nair et al. (2023)	1	0.33	0.33	1	0.33	0	0.66	0.66	4.31
Asare et al. (2023)	1	1	1	1	1	1	1	1	8
Tony et al. (2023)	0.66	0.66	0.66	0.33	0.66	1	0.66	0.33	4.96
Jesse et al. (2023)	1	1	1	1	0.66	1	1	0.66	7.31
He and Vechev (2023)	1	1	1	1	1	0.33	1	0.66	6.99
Sandoval et al. (2023)	1	1	1	1	1	1	1	1	8
Liguori et al. (2023)	1	0.66	0.66	0	0	1	1	1	5.32
Niu et al. (2023)	1	1	1	1	1	0.33	1	1	7.33

Articles that did not pass the minimum threshold (four points) are not included.

### 4.3 Final sample

The quality check discarded three papers, which left us with 19 as a final sample, as seen in Table 9. The first article published in this sample was in 2022 and the number of publications has been increasing every year. This situation is not surprising, as generative AI has risen in popularity in 2020 and has expanded into widespread knowledge with the release of ChatGPT 3.5.

**Table 9:** Sample of papers, with the main information of interest (^†^means no parameter or base model was specified in the article).

**References**	**Topic**	**AI-base model/parameter^†^ (Organization)**	**Language(s)**	**Vulnerability(ies)**
Pearce et al. (2022)	Insecure code generation	Codex Copilot^†^ (OpenAI)	C, Python and Verilog	MITRE's CWE Top 25 (2021)
Botacin (2023)	Malware code generation	ChatGPT- GPT3 (OpenAI)	C	Malware samples
Tony et al. (2022)	Cryptographic API calls	DeepAPI (DeepAI), DeepAPI-plusSec and DeepAPI-onlySec (both created by the authors)	Java	Misusing cryptographic API calls sequences
Pearce et al. (2023)	Insecure software code generation	Codex family—code-cushman-001 and code-davinci-001 and code-davinci-002 (OpenAI), J1-jumbo - 178B and J1-large—7.8B (AI21), Polycoder—2.7B (Xu et al., 2022), Gpt2-csrc—774M (locally trained model by the authors)	C, Python and Verilog	CWEs: [Software] 787, 089, 079, 125, 020, 416, 476, 119 and 732; [Hardware] 1271 and 1234
Nair et al. (2023)	Insecure hardware code generation	ChatGPT^†^ (OpenAI)	Verilog	Hardware Design CWE: 1194, 1221, 1224, 1234, 1245, 1254, 1255, 1271, 1276, 1280, 1298
Jha and Reddy (2023)	Exploit	Agnostic, but tested in CodeT5^†^ (Salesforce, Wang et al., 2021), CodeBert - 125 (Feng et al., 2020), GraphCodeBERT^†^ (Guo et al., 2021), RoBERTa^†^ (Facebook, Liu et al., 2019)	C#, Java, Python and PHP	Generation of adversarial code by attacking the vulnerable token
Sandoval et al. (2023)	HCI for security	Codex family-code-cushman-001 and code-davinci-001 and code-davinci-002 (OpenAI)	C	Impact of AI assistance in secure code production
Pa Pa et al. (2023)	Malware code generation	Auto-GPT-GPT-3.5-turbo and gpt-4-32k (Significant Gravitas), ChatGPT- GPT-3.5-turbo and text-davinci-003 (OpenAI)	C++, Python and GO	Jailbreaking
He and Vechev (2023)	Hardening and downgrading security code (controlled code generation)	CodeGen - 350M, 2.7B and 6.1B (Salesforce, Nijkamp et al., 2023), Codex Copilot^†^ (OpenAI)	C, C++ and Python	Different MITRE's Top-25: 022, 078, 079, 089, 119, 125, 190, 416, 476, 501, 732, 787, and 798.
Jesse et al. (2023)	Software bugs generation	Codex family—cushman-codex 12B, davinci-codex 175B (OpenAI), CodeGen - 350M, 2B, 6B, and 16B (Salesforce, Nijkamp et al., 2023) and PolyCoder- 160M, 0.4B and 2.6B (Xu et al., 2022)	Java	Simple stupid bugs generation comparison between AI models
Wu et al. (2023)	AI code generation fixing vulnerabilities	Codex family-davinci-002 (OpenAI), CodeT5-770M (Salesforce, Wang et al., 2021), CodeGen-6B (Salesforce, Nijkamp et al., 2023), PLBART-400M (Ahmad et al., 2021) and InCoder-6B (Fried et al., 2022)	Java	Capabilities and quality of the generated code for fixing security issues
Asare et al. (2023)	HCI for security	Codex Copilot^†^ (OpenAI)	C and C++	28 CWE from Big-Vul: 020, 119, 190, 284, 399, 476 664, 666, 682, 691, 693, 707 and 710 (listed in the paper)
Niu et al. (2023)	Exploit	Codex Copilot^†^ (OpenAI), CodeParrot - GPT-2 1.5B (HuggingFaces (2022)), PolyCoder - GPT-2 2.7B (Xu et al. (2022)) and StarCoder - 15.5B (Li et al. (2023))	Python	Membership inference attack for personal data leaks
Perry et al. (2023)	HCI for security	Codex-davinci-002 (OpenAI)	C, Java and Python	Code security for encryption, signing messages, sandbox directory, and SQL injection
Storhaug et al. (2023)	Insecure software code generation	GPT-J-6B (Eleuther-AI)	Solidity	Avoiding smart contract vulnerable code generation
Jia et al. (2023)	Adversarial code generation	ContraCode^†^ (Jain et al., 2021) and M1^†^ (Henkel et al., 2022)	Java and Python	Code-generation AI models manipulation by “adversarial inputs”
Tony et al. (2023)	Insecure software code generation	Codex-code-davinci-002 (OpenAI)	C and Python	MITRE's CWE Top 25 (2021)
Liguori et al. (2023)	Malware code generation	Seq2Seq (Britz et al., 2017) and CodeBERT-RoBERTA (Microsoft)	Assembly, Java and Python	Optimization of AI code generation models for malware production
Siddiq et al. (2022)	Insecure software code generation	Codex Copilot^†^ (OpenAI) and GPT-Code-Clippy^†^ (Multiple authors, 2021)	Python	Code smells in AI generated code.

## 5 Discussion

### 5.1 About AI models comparisons and methods for investigation

Almost the majority (14 papers—73%) of the papers research at least one OpenAI model, Codex being the most popular option. OpenAI owns ChatGPT, which was adopted massively by the general public. Hence, it is not surprising that most articles focus on OpenAI models. However, other AI models from other organizations are also studied, Salesforce's CodeGen and CodeT5, both open-source, are prime examples. Similarly, Xu et al. (2022) Polycoder was a popular selection in the sample. Finally, different authors benchmarked in-house AI models and popular models. For example, papers such as Tony et al. (2022) with DeepAPI-plusSec and DeepAPI-onlySec and Pearce et al. (2023) with Gpt2-csrc. Figure 3 shows the LLM instances researched by two or more articles grouped by family.

As the different papers researched different vulnerabilities, it remains difficult to compare the results. Some articles researched specific CWE, other MITRE Top-25, the impact of AI in code, the quality of the code generated, and malware generation, among others. It was also challenging to find the same methodological approach for comparing results, and therefore, we can only infer certain tendencies. For this reason, future research could focus on generating a standardized approach and analyzing vulnerabilities to analyze the quality of security. Furthermore, it would be interesting to have more analysis between open-source and proprietary models.

Having stated this, two articles with similar approaches, topics, and vulnerabilities are Pearce et al. (2022, 2023). Both papers share authors, which can help explain the similarity in the approach. Both have similar conclusions on the security of the output of different OpenAI models: they can generate functional and safe code, but the percentage of this will vary between CWE and programming language (Pearce et al., 2022, 2023). For both authors, the security of the code generated in C was inferior to that in Python (Pearce et al., 2022, 2023). For example, Pearce et al. (2022) indicates that for Python, 39% of the code suggested is vulnerable and 50% for code in C. Pearce et al. (2023) highlights that the models they studied struggled with fixes for certain CWE, such as CWE-787 in C. So even though they compared different models of the OpenAI family, they produced similar results (albeit some models had better performance than others).

Based on the work of Pearce et al. (2023), when comparing OpenAI's models to others (such as the AI21 family, Polycoder, and GPT-csrc) in C and Python with CWE vulnerabilities, OpenAI's models would perform better than the rest. In the majority of the cases, code-davinci-002 would outperform the rest. Furthermore, when applying the AI models to other programming languages, such as Verilog, not all models (namely Polycoder and gpt2-csrc) supported it (Pearce et al., 2023). We cannot fully compare these results with other research articles, as they focused on different CWEs but identified tendencies. To name the difference,

- He and Vechev (2023) studies mainly CodeGen and mentions that Copilot can help with CWE-089,022 and 798. They do not compare the two AI models but compare CodeGen with SVEN. They use scenarios to evaluate CWE, adopting the method from Pearce et al. (2022). CodeGen does seem to provide similar tendencies as Pearce et al. (2022): certain CWE appeared more recurrently than others. For example, comparing with Pearce et al. (2022) and He and Vechev (2023), CWE-787, 089, 079, and 125 in Python and C appeared in most scenarios at a similar rate.^4^
  This data shows that even OpenAI's and CodeGen models have similar outputs. When He and Vechev (2023) present the “overall security rate” at different temperatures of CodeGen, they have equivalent security rates: 42% of the code suggested being vulnerable in He and Vechev (2023) vs. a 39% in Python and 50% in C in Pearce et al. (2022).
- Nair et al. (2023) also studies CWE vulnerabilities for Verilog code. Both Pearce et al. (2022, 2023) also analyze Verilog in OpenAI's models, but with very different research methods. Furthermore, their objectives are different: Nair et al. (2023) focuses on prompting and how to modify prompts for a secure output. What can be compared is that Nair et al. (2023) and Pearce et al. (2023) highlight the importance of prompting.
- Finally Asare et al. (2023) also studies OpenAI from a very different perspective: the human-computer interaction (HCI). Therefore, we cannot compare the study results of Asare et al. (2023) with Pearce et al. (2022, 2023).

Regarding malware code generation, both Botacin (2023) and Pa Pa et al. (2023) OpenAI's models, but different base-models. Both conclude that AI models can help generate malware but to different degrees. Botacin (2023) indicates that ChatGPT cannot create malware from scratch but can create snippets and help less-skilled malicious actors with the learning curve. Pa Pa et al. (2023) experiment with different jailbreaks and suggest that the different models can create malware, up to 400 lines of code. In contrast, Liguori et al. (2023) researchers Seq2Seq and CodeBERT and highlight the importance for malicious actors that AI models output correct code if not their attack fails. Therefore, human review is still necessary to fulfill the goals of malicious actors (Liguori et al., 2023). Future work could benefit from comparing these results with other AI code generation models to understand if they have similar outputs and how to jailbreak them.

The last element we can compare is the HCI aspects, specifically Asare et al. (2023), Perry et al. (2023), and Sandoval et al. (2023), who all researched on C. Both Asare et al. (2023) and Sandoval et al. (2023) agree that AI code generation models do not seem to be worse, if not the same, in generating insecure code and introducing vulnerabilities. In contrast, Perry et al. (2023) concludes that developers who used AI assistants generated more insecure code—although this is inconclusive for the C language—as these developers believed they had written more secure code. Perry et al. (2023) suggest that there is a relationship between how much trust there is between the AI model and the security of code. All three agree that AI assistant tools should not be used carefully, particularly between non-experts (Asare et al., 2023; Perry et al., 2023; Sandoval et al., 2023).

### 5.2 New exploits

Firstly, Niu et al. (2023) hand-crafted prompts that seemed could leak personal data, which yielded 200 prompts. Then, they queried each of these prompts, obtaining five responses per prompt, giving 1,000 responses. Two authors then looked through the outputs to identify if the prompts had leaked personal data. The authors then improved these with the identified prompts. They tweaked elements such as context, pre-fixing or the natural language (English and Chinese), and meta-variables such as prompt programming language style for the final data set.

With the final set of prompts, the model was queried for privacy leaks. B efore querying the model, the authors also tuned specific parameters, such as temperature. “Using the BlindMI attack allowed filtering out 20% of the outputs, with the high recall ensuring that most of the leakages are classified correctly and not discarded (Niu et al., 2023).” Once the outputs had been labeled as members, a human checked if they contained “sensitive data” (Niu et al., 2023). The human could categorize such information as targeted leak, indirect leak, or uncategorized leak.

When applying the exploit to Codex Copilot and verifying with GitHub, it shows there is indeed a leakage of information (Niu et al., 2023). 2.82% of the outputs contained identifiable information such as address, email, and date of birth; 0.78% private information such as medical records or identities; and 0.64% secret information such as private keys, biometric authentication or passwords (Niu et al., 2023). The instances in which data was leaked varied; specific categories, such as bank statements, had much lower leaks than passwords, for example Niu et al. (2023). Furthermore, most of the leaks tended to be indirect rather than direct. This finding implies that “the model has a tendency to generate information pertaining to individuals other than the subject of the prompt, thereby breaching privacy principles such as contextual agreement (Niu et al., 2023).”

Their research proposes a scalable and semi-automatic manner to leak personal data from the training data in a code-generation AI model. The authors do note that the outputs are not verbatim or memorized data.

To achieve this, He and Vechev (2023) curated a dataset of vulnerabilities from CrossVul (Nikitopoulos et al., 2021) and Big-Vul (Fan et al., 2020), which focuses in C/C++ and VUDENC (Wartschinski et al., 2022) for Python. In addition, they included data from commits from GitHub, taking into special consideration that they were true commits, avoiding that SVEN learns “undesirable behavior.” At the end, they target 9 CWES from MITRE Top 25.

Through benchmarking, they evaluate SVEN output's security (and functional) correctness against CodeGen (350M, 2.7B, and 6.1B). They follow a scenario-based approach “that reflect[s] real-world coding (He and Vechev, 2023),” with each scenario targeting one CWE. They measure the security rate, which is defined as “the percentage of secure programs among valid programs (He and Vechev, 2023).” They set the temperature at 0.4 for the samples.

Their results show that SVEN can significantly increase and decrease (depending on the controlled generation output) the code security score. “CodeGen LMs have a security rate of ≈60%, which matches the security level of other LMs [...] SVEN_*sec*_ significantly improves the security rate to >85%. The best-performing case is 2.7B, where SVENsec increases the security rate from 59.1 to 92.3% (He and Vechev, 2023).” Similar results are obtained for SVEN_*vul*_ with the “security rate greatly by 23.5% for 350M, 22.3% for 2.7B, and 25.3% for 6.1B (He and Vechev, 2023)”.^5^ When analyzed per CWE, in almost all cases (except CWE-416 language C) SVEN_*sec*_ increases the security rate. Finally, even when tested with 4 CWE that were not included in the original training set of 9, SVEN had positive results.

Although the authors aim at evaluating and validating SVEN, as an artifact for cybersecurity, they also recognize its potential use as a malicious tool. They suggest that SVEN can be inserted in open-source projects and distributed (He and Vechev, 2023). Future work could focus on how to integrate SVEN—or similar approaches—as plug-ins into AI code generations, to lower the security of the code generated. Furthermore, replication of this approach could raise security alarms. Other research can focus on seeking ways to lower the security score while keeping the functionality and how it can be distributed across targeted actors.

They benchmark CodeAttack against the TextFooler and BERT-Attack, two other adversarial attacks in three tasks: code translation (translating code between different programming languages, in this case between C# and Java), code repair (fixes bugs for Java) and code (a summary of the code in natural language). The authors also applied the benchmark in different AI models (CodeT5, CodeBERT, GraphCode-BERT, and RoBERTa) in different programming languages (C#, Java, Python, and PHP). In the majority of the tests, CodeAttack had the best results.

### 5.3 Performance per programming language

**Figure 2:**
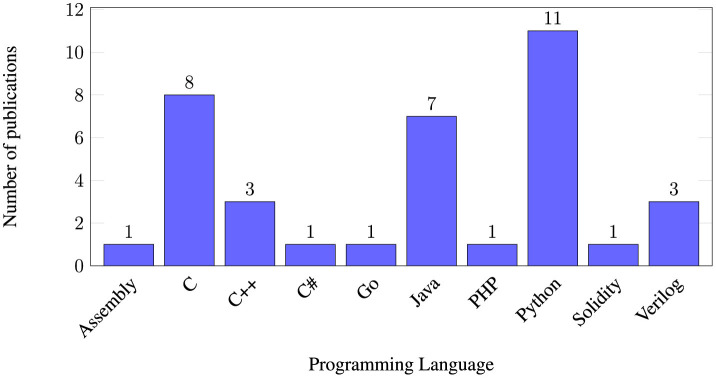
Number of articles that research specific programming languages. An article may research 2 or more programming languages.

Different programming languages are studied. Python and the C family are the most common languages, including C, C++, and C# (as seen in Figure 2). To a lesser extent, Java and Verilog are tested. Finally, specific articles would study more specific programming languages, such as Solidity, Go or PHP. Figure 2 offers a graphical representation of the distribution of the programming languages.

**Figure 3:**
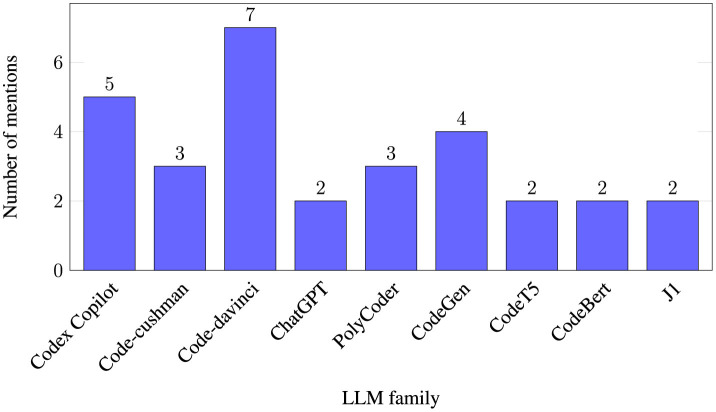
Number of times each LLM instance was researched by two or more articles, grouped by family. One paper might study several instances of the same family (e.g., Code-davinci-001 and Code-davinci-002), therefore counting twice. Table 9 offers details on exactly which AI models are studied per article.

#### 5.3.1 Python

Python is the second most used programming language^6^ as of today. As a result most publicly-available training corpora include Python and it is therefore reasonable to assume that AI models can more easily be tuned to handle this language (Pearce et al., 2022, 2023; Niu et al., 2023; Perry et al., 2023). Being a rather high level, interpreted language, Python should also expose a smaller attack surface. As a result, AI-generated Python code has fewer avenues to cause issues to begin with, and this is indeed backed up by evidence (Pearce et al., 2022, 2023; Perry et al., 2023).

In spite of this, issues still occur: Pearce et al. (2022) experimented with 29 scenarios, producing 571 Python programs. Out of these, 219 (38.35%) presented some kind of Top-25 MITRE (2021) vulnerability, with 11 (37.92%) scenarios having a top-vulnerable score. Unaccounted in these statistics are the situations where generated programs fail to achieve functional correctness (Pearce et al., 2023), which could yield different conclusions.^7^

Pearce et al. (2023), building from Pearce et al. (2022), study to what extent post-processing can automatically detect and fix bugs introduced during code generation. For instance, on CWE-089 (SQL injection) they found that “29.6% [3197] of the 10,796 valid programs for the CWE-089 scenario were repaired” by an appropriately-tuned LLM (Pearce et al., 2023). In addition, they claim that AI models can generate bug-free programs without “additional context (Pearce et al., 2023).”

It is however difficult to support such claims, which need to be nuanced. Depending on the class of vulnerability, AI models varied in their ability in producing secure Python code (Pearce et al., 2022; He and Vechev, 2023; Perry et al., 2023; Tony et al., 2023). Tony et al. (2023) experimented with code generation from natural language prompts, findings that indeed, Codex output included vulnerabilities. In another research,Copilot reports only rare occurences of CWE-079 or CWE-020, but common occurences of CWE-798 and CWE- 089 (Pearce et al., 2022). Pearce et al. (2022) report a 75% vulnerable score for scenario 1, 48% scenario 2, and 65% scenario 3 with regards to CWE-089 vulnerability (Pearce et al., 2022). In February 2023, Copilot launched a prevention system for CWEs 089, 022, and 798 (He and Vechev, 2023), the exact mechanism of which is unclear. At the time of writing it falls behind other approaches such as SVEN (He and Vechev, 2023).

Perhaps surprisingly, there is not much variability across different AI models: CodeGen-2.7B has comparable vulnerability rates (He and Vechev, 2023), with CWE-089 still on top. CodeGen-2.7B also produced code that exhibited CWE-078, 476, 079, or 787, which are considered more critical.

One may think that using AI as an assistant to a human programmer could alleviate some of these issues. Yet evidence points to the opposite: when using AI models as pair programmers, developers consistently deliver more insecure code for Python (Perry et al., 2023). Perry et al. (2023) led a user-oriented study on how the usage of AI models for programming affects the security and functionality of code, focusing on Python, C, and SQL. For Python, they asked participants to write functions that performed basic cryptographic operations (encryption, signature) and file manipulation.^8^ They show a statistically significant difference between subjects that used AI models (experimental group) and those that did not (control group), with the experimental group consistently producing less secure code (Perry et al., 2023). For instance, for task 1 (encryption and decryption), 21% of the responses of the experiment group was secure and correct vs. 43% of the control group (Perry et al., 2023). In comparison, 36% of the experiment group provided insecure but correct code, compared to 14%.

Even if AI models produce on occasion bug-free and secure code, evidence points out that it cannot be guaranteed. In this light, both Pearce et al. (2022, 2023) recommend deploying additional security-aware tools and methodologies whenever using AI models. Moreover, Perry et al. (2023) suggests a relationship between security awareness and trust in AI models on the one hand, and the security of the AI-(co)generated code.

Another point of agreement in our sample is that prompting plays a crucial role in producing vulnerabilities, which can be introduced or avoided depending on the prompt and adjustment of parameters (such as temperature). Pearce et al. (2023) observes that AI models can generate code that repairs the issue when they are given a suitable repair prompt. Similarly, Pearce et al. (2022) analyzed how meta-type changes and comments (documentation) can have varying results over the security (Pearce et al., 2022). An extreme example is the difference between an SQL code generated with different prompts: the prompt “adds a separate non-vulnerable SQL function above a task function” (identified as variation C-2, as it is a code change) would never produce vulnerable code whereas “adds a separate vulnerable SQL function above the task function” (identified as variation C-3) returns vulnerable code 94% of the time (Pearce et al., 2022). Such results may not be surprising if we expect the AI model to closely follow instructions, but suffice to show the effect that even minor prompt variations can have on security.

Lastly, Perry et al. (2023) observe in the experimental group a relationship between parameters of the AI model (such as temperature) and code quality. They also observe a relationship between education, security awareness, and trust (Perry et al., 2023). Because of this, there could be spurious correlations in their analysis, for instance the variable measuring AI model parameters adjustments could be, in reality, measuring education or something else.

On another security topic, Siddiq et al. (2022) study code and security “smells.” Smells are hints, not necessarily actual vulnerabilities, but they can open the door for developers to make mistakes that lead to security flaws that attackers exploit. Siddiq et al. (2022) reported on the following CWE vulnerabilities: 078,703,330. They have concluded that bad code patterns can (and will) leak to the output of models, and code generated with these tools should be taken with a “grain of salt” (Siddiq et al., 2022). Furthermore, identified vulnerabilities may be severe (not merely functional issues) (Siddiq et al., 2022). However, as they only researched OpenAI's AI models, their conclusion may lack external validity and generalization.

Finally, some authors explore the possibility to use AI models to deliberately produce malicious code (He and Vechev, 2023; Jha and Reddy, 2023; Jia et al., 2023; Niu et al., 2023). It is interesting to the extent that this facilitates the work of attackers, and therefore affects cybersecurity as a whole, but it does not (in this form at least) affect the software development process or deployment per se, and is therefore outside of the scope of our discussion.

#### 5.3.2 C

The C programming language is considered in 10 (52%) papers of our final sample, with C being the most common, followed by C++ and C#. Unlike Python, C is a low-level, compiled language, that puts the programmer in charge of many security-sensitive tasks (such as memory management). The vast majority of native code today is written in C.^9^

The consensus is that AI generation of C programs yields insecure code (Pearce et al., 2022, 2023; He and Vechev, 2023; Perry et al., 2023; Tony et al., 2023), and can readily be used to develop malware (Botacin, 2023; Liguori et al., 2023; Pa Pa et al., 2023). However, it is unclear whether AI code generation introduce more or new vulnerabilities compared to humans (Asare et al., 2023; Sandoval et al., 2023), or to what extent they influence developers' trust in the security of the code (Perry et al., 2023).

Multiple authors report that common and identified vulnerabilities are regularly found in AI-generated C code (Pearce et al., 2022, 2023; Asare et al., 2023; He and Vechev, 2023; Perry et al., 2023; Sandoval et al., 2023). Pearce et al. (2022) obtained 513 C programs, 258 of which (50.29% ) had a top-scoring vulnerability. He and Vechev (2023) provides a similar conclusion.

About automated code-fixing, Asare et al. (2023) and Pearce et al. (2023) report timid scores, with only 2.2% of C code for CWE-787.

On the question of human- vs. AI-generated code, Asare et al. (2023) used 152 scenarios to conclude that AI models make in fact fewer mistakes. Indeed, when prompted with the same scenario as a human, 33% cases suggested the original vulnerability, and 25% provided a bug-free output. Yet, when tested on code replication or automated vulnerability fixing, the authors do not recommend the usage of a model by non-experts. For example, in code replication, AI models would always replicate code regardless of whether it had a vulnerability, and CWE-20 would consistently be replicated (Asare et al., 2023).

Sandoval et al. (2023) experimentally compared the security of code produced by AI-assisted students to the code generated by Codex. They had 58 participants and studied memory-related CWE, given that they are in the Top-25 MITRE list (Sandoval et al., 2023). Although there were differences between groups, these were not bigger than 10% and would differ between metrics (Sandoval et al., 2023). In other words, depending on the chosen metric, sometimes AI-assisted subjects perform better in security and vice versa (Sandoval et al., 2023). For example, CWE-787 was almost the same for the control and experimental groups, whereas the generated Codex code was prevalent. Therefore, they conclude that the impact on “cybersecurity is less conclusive than the impact on functionality (Sandoval et al., 2023).” Depending on the security metric, it may be beneficial to use AI-assisted tools, which the authors recognize goes against standard literature (Sandoval et al., 2023). They go so far as to conclude that there is “no conclusive evidence to support the claim LLM assistant increase CWE incidence in general, even when we looked only at severe CWEs (Sandoval et al., 2023).”

Regarding AI-assisted malware generation, there seems to be fundamental limitations preventing current AI models from writing self-contained software from scratch (Botacin, 2023; Liguori et al., 2023; Pa Pa et al., 2023), although it is fine for creating smaller blocks of code which, strung together, produce a complete malware (Botacin, 2023). It is also possible to bypass models' limitations by leveraging basic obfuscation techniques (Botacin, 2023). Pa Pa et al. (2023) experiment prompts and jailbreaks in ChatGPT to produce code (specifically, fileless malware for C++), which was only provided with 2 jailbreaks they chose. While Liguori et al. (2023) reflect on how to best optimize AI-generating tools to assist attackers in producing code, as failure or incorrect codes means the attack fails.

Over CWE, Top MITRE-25 is a concern across multiple authors (Pearce et al., 2022, 2023; He and Vechev, 2023; Tony et al., 2023). CWE-787 is a common concern across articles, as it is the #1 vulnerability in the Top-25 MITRE list (Pearce et al., 2022; Botacin, 2023; He and Vechev, 2023). On the three scenarios experimented by Pearce et al. (2022), on average, ~34% of the output is vulnerable code. He and Vechev (2023) tested with two scenarios, the first receiving a security rate of 33.7% and the second one 99.6%. What was interesting in their experiment is that they were not able to provide lower security rates for SVEN_*vul*_ than the originals (He and Vechev, 2023). Other vulnerabilities had varying results but with a similar trend. Overall, it seems that the AI code generation models produce more vulnerable code compared to other programming languages, possibly due to the quality and type of data in the training data set (Pearce et al., 2022, 2023).

Finally, regarding human-computer interaction, Perry et al. (2023) suggests that subjects “with access to an AI assistant often produced more security vulnerabilities than those without access [...] overall.” However, they highlight that their difference is not statistically significant and inconclusive for the case they study in C. So even if the claim applies to Python, Perry et al. (2023) indicates this is not the case for the C language. Asare et al. (2023) and Sandoval et al. (2023), as discussed previously, both conclude that AI models do not introduce more vulnerabilities than humans into code. “This means that in a substantial number of scenarios we studied where the human developer has written vulnerable code, Copilot can avoid the detected vulnerability (Asare et al., 2023).”

#### 5.3.3 Java

Java^10^ is a high-level programming language that runs atop a virtual machine, and is today primarily used for the development of mobile applications. Vulnerabilities can therefore arise from programs themselves, calls to vulnerable (native) libraries, or from problems within the Java virtual machine. Only the first category of issues is discussed here.

In our sample, four articles (Tony et al., 2022; Jesse et al., 2023; Jha and Reddy, 2023; Wu et al., 2023) analyzed code generation AI models for Java. Each research focused on very different aspects of cyber security and they did not analyze the same vulnerabilities. Tony et al. (2022) investigated the dangers and incorrect of API calls for cryptographic protocols. Their conclusions is that generative AI might not be at all optimized for generating cryptographically secure code (Tony et al., 2022). The accuracy of the code generated was significantly lower on cryptographic tasks than what the AI is advertised to have on regular code (Tony et al., 2022).

Jesse et al. (2023) experiments with generating single stupid bugs (SStuB) with different AI models. They provide six main findings, which can be summarized as: AI models propose twice as much SSTuB as correct code. However, they also seem to help with other SStuB (Jesse et al., 2023).^11^ One of the issues with SStuBs is that “where Codex wrongly generates simple, stupid bugs, these may take developers significantly longer to fix than in cases where Codex does not (Jesse et al., 2023).” In addition, different AI models would behave differently over the SStuBs generated (Jesse et al., 2023). Finally, Jesse et al. (2023) found that commenting on the code leads to fewer SStuBs and more patches, even if the code is misleading.

Wu et al. (2023) analyze and compare (1) the capabilities of different LLMs and fine-tuned LLMs and automated program repair (APR) techniques for repairing vulnerabilities in Java; (2) proposes VJBench and VJBench-trans as a “new vulnerability repair benchmark;” (3) and evaluates the studied AI models on their proposed VJBench and VJBench-trans. VJBench aims to extend the work of Vul4J and thus proposes 42 vulnerabilities, including 12 new CWEs that were not included in Vul4J (Wu et al., 2023). Therefore, their study assessed 35 vulnerabilities proposed by Vul4J and 15 by the authors (Wu et al., 2023). On the other hand, VJBench-trans is composed of “150 transformed Java vulnerabilities (Wu et al., 2023).” Overall, they concluded that the AI models fix very few Java vulnerabilities, with Codex fixing 20.4% of them (Wu et al., 2023). Indeed, “large language models and APR techniques, except Codex, only fix vulnerabilities that require simple changes, such as deleting statements or replacing variable/method names (Wu et al., 2023).” Alternatively, it seems that fine-tuning helps the LLMs improve the task of fixing vulnerabilities (Wu et al., 2023).

However, four APR and nine LLMs did not fix the new CWEs introduced by VJBench (Wu et al., 2023). Some CWEs that are not tackled are “CWE-172 (Encoding error), CWE-325 (Missing cryptographic step), CWE-444 (HTTP request smuggling; Wu et al., 2023),” which can have considerable cybersecurity impacts. For example, CWE-325 can weaken a cryptographic protocol, thus lowering the security capacity. Furthermore, apart from Codex, the other AI models and APR studied did not apply complex vulnerability repair but would focus on “simple changes, such as deletion of a statement (Wu et al., 2023).”

Jia et al. (2023) study the possibility that a code-generation AI model is manipulated by “adversarial inputs.” In other words, the user inputs designed to trick the model into either misunderstanding code, or producing code that behaves in an adversarially-controlled way. They tested Claw, M1 and ContraCode both in Python and Java for the following tasks: code summarization, code completion and code clone detection (Jia et al., 2023).

Finally, Jha and Reddy (2023) proposes *CodeAttack*, which is implemented in different programming languages, including Java.^12^ When tested in Java, their results show that 60% of the adversarial code generated is syntactically correct (Jha and Reddy, 2023).

#### 5.3.4 Verilog

Verilog is a hardware-description language. Unlike other programming languages discussed so far, its purpose is not to describe software but to design and verify of digital circuits (at the register-transfer level of abstraction).

The articles that researched Verilog generally conclude that the AI models they researched are less efficient in this programming language than Python or C (Pearce et al., 2022, 2023; Nair et al., 2023). Different articles would research different vulnerabilities, with two specific CWEs standing out: 1271 and 1234. Pearce et al. (2022) summarizes the difficulty of defining which vulnerability to study from the CWE for Verilog, as there is no Top 25 CWE for hardware. Hence, their research selected vulnerabilities that could be analyzed (Pearce et al., 2022). This situation produces difficulties in comparing research and results, as different authors can select different focuses. The different approaches to vulnerabilities in Verilog can be seen in Table 9, where only two CWE are common across all studies (1271 and 1234), but others such as 1221 (Nair et al., 2023) or 1294 (Pearce et al., 2022) are researched by one article.

Note that unlike software vulnerabilities, it is much harder to agree on a list of the most relevant hardware vulnerabilities, and to the best of our knowledge there is no current consensus on the matter today.

Regarding the security concern, both Pearce et al. (2022, 2023), studying OpenAI, indicated that in general these models struggled to produce correct, functional, and meaningful code, being less efficient over the task. For example, Pearce et al. (2022) generates “198 programs. Of these, 56 (28.28%) were vulnerable. Of the 18 scenarios, 7 (38.89 %) had vulnerable top-scoring options.” Pearce et al. (2023) observes that when using these AI models to generate repair code, firstly, they had to vary around with the temperature of the AI model (compared to C and Python), as it produced different results. Secondly, they conclude that the models behaved differently with Verilog vs. other languages and “seemed [to] perform better with less context provided in the prompt (Pearce et al., 2023).” The hypothesis on why there is a difference between Verilog and other programming languages is because there is less training data available (Pearce et al., 2022).

### 5.4 Mitigation strategies

There have been several attempts, or suggestions, to mitigate the negative effects on security when using AI to code. Despite reasonable, not all are necessarily effective, as we discuss in the remainder of this section. Overall, the attempts we have surveyed discuss how modify the different elements that can affect the quality of the AI models or the quality of the user control over the AI-generated code. Table 10 summarizes the suggested mitigation strategies.

**Table 10:** Summary of the mitigation strategies.

**Mitigation strategy**	**Main points**
Dataset	• Better quality dataset • Adding different programming languages • Trade-offs between the size of the training set and the ability to generate code and generalize.
Training procedure	• Stricter training regime on syntactic and (some degree of) semantic correctness of the output • Fine-tunning with carefully curated data; although there are divergent views on this topic.
Generation procedure	• Context of prompt is important • Guidelines or best practices for prompting • Limitations or safeguarding prompts (and jailbreaking) • Post-processing the outputs.
Integration of AI-generated code into software	• Procedures and process for security check of the suggested code • Keeping a level of mistrust toward AI code generation tools.
End-user education	• Education on the limitations of AI code generation models • Human-oversight • Possible design changes in the user interface

#### 5.4.1 Dataset

Part of the issue is that LLMs are trained on code that is itself ripe with vulnerabilities and bad practice. As a number of the AI models are not open-source or their training corpora is no available, different researchers hypothesize that the security issue arise from the training dataset (Pearce et al., 2022). Adding datasets that include different programming languages with different vulnerabilities may help reduce the vulnerabilities in the output (Pearce et al., 2022). This is why, to mitigate the problems with dataset security quality, He and Vechev (2023) manually curated the training data for fine-tuning, which improved the output performance against the studied CWE.

By carefully selecting training corpora that are of higher quality, which can be partially automated, there is hope that fewer issues would arise (He and Vechev, 2023). However, a consequence of such a mitigation is that the size of the training set would be much reduced, which weakens the LLM's ability to generate code and generalize (Olson et al., 2018). Therefore one may expect that being too picky with the training set would result, paradoxically, in a reduction in code output quality. A fully fledged study of this trade-off remains to be done.

#### 5.4.2 Training procedure

During the training process, LLMs are scored on their ability to autoencode, that is, to accurately reproduce their input (in the face of a partially occulted input). In the context of natural language, minor errors are often acceptable and almost always have little to no impact on the meaning or understanding of a sentence. Such is not the case for code, which can be particularly sensitive to minor variations, especially for low-level programming languages. A stricter training regimen could score an LLM based not only on syntactic correctness, but on (some degree of) semantic correctness, to limit the extent to which the model wanders away from a valid program. Unfortunately, experimental data from Liguori et al. (2023) suggests that currently no single metric succeeds at that task.

Alternatively, since most LLMs today come pre-trained, a better fine-tuning step could reduce the risks associated with incorrect code generation. He and Vechev (2023) took this approach and had promising results in the CWE they investigated. However, there is conflicting evidence. Evidence from Wu et al. (2023) seems to indicate that this approach is inherently limited to fixing a very narrow, and simple class of bugs. More studies analyzing the impact of fine-tuning models with curated security datasets are needed to assess the impact of this mitigation strategy.

#### 5.4.3 Generation procedure

Code quality is improved by collecting more *context* that the user typically provides through their prompts (Pearce et al., 2022; Jesse et al., 2023). The ability to use auxiliary data, such as other project files, file names, etc. seems to explain the significant difference in code acceptation between GitHub Copilot and its bare model OpenAI Codex. The exploration of creating guidelines and best practices on how to do prompts effectively may be interesting. Nair et al. (2023) explored the possibility of creating prompt strategies and techniques for ChatGPT that would output secure code.

From an adversarial point of view, Niu et al. (2023) provides evidence of the impact of *context* and prompts for exploiting AI models. There are ongoing efforts to limit which prompts are accepted by AI systems by safeguarding them (Pa Pa et al., 2023). However, Pa Pa et al. (2023) showed—with mixed results—how to bypass these limitations, what is called “jailbreaking.” Further work on this area is needed as a mitigation strategy and its effectiveness.

Independently, post-processing the output (SVEN is one example; He and Vechev, 2023) has a measurable impact on code quality, and is LLM-agnostic, operating without the need for re-training nor fine-tuning. Presumably, non-LLM static analyzers or linters may be integrated as part of the code generation procedure to provide checks along the way and avoid producing code that is visibly incorrect or dangerous.

#### 5.4.4 Integration of AI-generated code into software

Even after all the technical countermeasures have been taken to avoid producing code that is obviously incorrect, there remains situations where AI-generated programs contain (non-obvious) vulnerabilities. To a degree, such vulnerabilities could also appear out of human-generated code, and there should in any case be procedures to try and catch these as early as possible, through unit, functional and integration testing, fuzzing, or static analysis. Implementation of security policies and processes remains vital.

However AI models are specifically trained to produce code that *looks* correct, meaning that their mistakes may be of a different nature or appearance than those typically made by human software programmers, and may be harder to spot. At the same time, the very reason why code generation is appealing is that it increases productivity, hence the amount of code in question.

It is therefore essential that software developers who rely on AI code generation keep a level of mistrust with regards to these tools (Perry et al., 2023). It is also likely that code review methodologies should be adjusted in the face of AI-generated code to look for the specific kind of mistakes or vulnerabilities that this approach produces.

#### 5.4.5 End-user education

One straightforward suggestion is educating users to assess the quality of software generated with AI models. Among the works we have reviewed, we found no studies that specifically discuss the quality and efficacy of this potential mitigation strategy, so we can only speculate about it from related works. For instance, Moradi Dakhel et al. (2023) compares the code produced by human users with the code generated by GitHub Copilot. The study is not about security. It is about the correctness of the implementation of quite well-known algorithms. Still, human users—students with an education in algorithms—performed better than their AI counterparts, but the buggy solutions generated by Copilot were easily fixable by the users. Relevantly, the AI-generated bugs were more easily recognizable and fixable than those produced by other human developers performing the same task.

This observation suggests that using AI could help write code faster for programmers skilled in debugging and that this task should not hide particular complexity for them. As Chen et al. (2021) suggested, “human oversight and vigilance is required for safe use of code generation systems like Codex.” However, removing obvious errors from buggy implementations of well-known algorithms is not the same as spotting security vulnerabilities: the latter task is complex and error-prone, even for experts. And here we speculate that if AI-generated flaws are naïve, programmers can still have some gain from using AI if they back up coding with other instruments used in security engineering (e.g., property checking, code inspection, and static analysis). Possible design changes or decision at the user interfaces may also have an impact. However, we have no evidence of whether our speculative idea can work in practice. The question remains open and calls for future research.

## 6 Threats to validity and future work

Previous literature Wohlin et al. (2013) and Petersen et al. (2015) have identified different reliability and validity issues in systematic literature reviews. One of the first elements that needs to be noted is the sample of papers. As explained by Petersen et al. (2015), the difference between systematic mapping studies and systematic literature reviews is the sample's representativeness; mappings do not necessarily need to obtain the whole universe of papers compared with literature reviews. Nevertheless, previous research has found that even two exact literature reviews on the same subject do not have the same sample of papers, affecting it. Consequently, to increase the reliability, we identified the PICO of our research and used golden standard research methods for SLR, such as Kitchenham and Charters (2007). This strategy helps us develop different strings for the databases tested to obtain the most optimal result. Furthermore, aiming to obtain a complete sample, we followed a forward snowballing of the whole sample obtained in the first round, as suggested by Wohlin et al. (2013) and Petersen et al. (2015).

However, there may still be reliability issues with the sample. Firstly, the amount of ongoing publications on the subjects increases daily. Therefore, the total number would increase depending on the day the sample was obtained. Furthermore, some research on open-source platforms (such as ArXiV) did not explicitly indicate if it was peer-reviewed. Hence, the authors manually checked whether it was accepted at a peer-review venue. This is why we hypothesize that the snowballing phase provided many more papers, as these had yet to be indexed in the databases and were only available at open-source platforms. Therefore, the final sample of this research may increase and change depending on the day the data was gathered.

In addition, the sample may differ based on the definition of “code generation.” For this research and as explained in Section 4 , we worked around the idea that AI models should suggest code (working or not). Some papers would fall under our scope in some cases, even if the main topic were “verification and validation,” as the AI tools proposed for this would suggest code. Hence, we focus not only on the development phase of the SDLC but also on any phase that suggests code. Different handling of “code generation” may provide different results.

On another note, the background and expertise of the researchers affect how papers are classified and information is extracted (Wohlin et al., 2013). In this manner, in this research, we used known taxonomies and definitions for classification schemes, such as Wieringa et al. (2006) for the type of research or MITRE's Top Vulnerabilities to identify which are the most commonly discussed risk vulnerabilities. The objective of using well-known classification schemes and methodologies is to reduce bias, as identified (Petersen et al., 2015). However, a complete reduction of bias cannot be ruled out.

Moreover, to fight authors' bias, every single article was reviewed, and data was extracted by at least two others, using a pairing strategy. If, due to time constraints, it was only reviewed by one author, the other author would review the work (Wohlin et al., 2013). If disagreements appeared at any phase – such as the inclusion/exclusion or data gathering – a meeting would be done and discussed (Wohlin et al., 2013). For example, in a couple of papers, Author #1 was unsure if it should be included or excluded based on the quality review, which was discussed with Author #4. Our objective in using a pairing strategy is to diminish authors' bias throughout the SLR.

On the analysis and comparison of the different articles, one threat to the validity of this SLR is that not all articles use the same taxonomy for vulnerabilities; they could not be classified under a single method. Some articles would research either MITRE's CWE or the Top-25, and others would tackle more specific vulnerabilities (such as jailbreaking, malware creation, SSB, and human programming). Therefore, comparing the vulnerabilities between the articles is, at best, complicated and, at worst, a threat to our conclusions. Given the lack of a classification scheme for the wide range of security issues tackled in our sample, we (1) tried to classify the papers based on the claims of the papers' articles; (2) we aimed at comparing based on the programming language used, and between papers researched similar subjects, such as MITRE's CWE. In this manner, we would not be comparing completely different subjects. As recognized by Petersen et al. (2015), the need for a classification scheme for specific subjects is a common challenge for systematic mapping studies and literature reviews. Nevertheless, future studies would benefit from a better classification approach if the sample permits.

We have provided the whole sample at: https://doi.org/10.5281/zenodo.10666386 for replication and transparency, with the process explained in detail. Each paper has details on why it was included/excluded, at which phase, and with details and/or comments to help readers understand and replicate our research. Likewise, we explained our research methods in as much detail as possible in the papers. Tangently, providing the details and open sources of the data helps us increase validity issues that may be present in this study.

Nonetheless, even when using well-known strategies both for the SLR and to mitigate known issues, we cannot rule out that there are inherent validity and reliability elements proper from all SLRs. We did our best efforts to mitigate these.

## 7 Conclusion

By systematically reviewing the state of the art, we aimed to provide insight into the question, “How does the code generation from AI models impact the cybersecurity of the software process?” We can confirm that there is enough evidence for us to say, unsurprisingly, that code generated by AI is not necessarily secure and it also contains security flaws. But, as often happens with AI, the real matter is not if AI is infallible but whether it performs better than humans doing the same task. Unfortunately, the conclusions we gathered from the literature diverge in suggesting whether AI-generated security artifacts should be cautiously approached, for instance, because of some particular severity or because they are tricky to spot. Indeed, some work reports of them as naïve and easily detectable, but the result cannot be generalized. Overall, there is no clear favor for one hypothesis over the other because of incomparable differences between the papers' experimental setups, data sets used for the training, programming languages considered, types of flaws, and followed experimental methodologies.

Generally speaking and regardless of the code production activity—whether for code generation from scratch, generating code repair, or even suggesting code—our analysis reveals that well-documented vulnerabilities in have been found in AI-suggested code, and this happened a non-negligible amount of times. And among the many, specific vulnerabilities, such as CWE MITRE Top-25, have received special attention in the current research and for a reason. For instance, CWE-787 and 089 received particular attention from articles, as they are part of the top 3 of MITRE CWE. Furthermore, the CWE security scores of generated code suggested by AI models would vary, with some CWEs being more prevalent than others.

Other works report on having found naïve bugs, easy to fix while other discovered malware code hidden between the benign lines, and other more reported an unjustified trust by human on the quality of the AI-generated code, an issue that raises concerns of a more socio-technical nature.

Similarly, when generated with AI support, different programming languages have different security performances. AI-generated Python code seemed to be more secure (i.e., have fewer bugs) than AI-generated code of the C family. Indeed, different authors have hypothesized that this situation is a consequence of the training data set and its quality. Verilog seems to suffer from similar shortcomings as C. When comparing the security of AI-generated Verilog to C or Python, the literature converges on reporting that the security of the former is worse. Once again, the suggested reason for the finding is that available training data sets for Verilog are smaller and of worse quality than those available for training AI models to generate C or Python code. In addition, there is no identified Top 25 CWE for Verilog. Java is another commonly studied programming language, with similar conclusions as once stated before. To a lesser extent, other programming languages that could be further studied were studied.

Looking at security exploits enabled by AI-generated code with security weaknesses, four different of them are those more frequently reported: SVEN, CodeAttack, and Codex Leaks. Such attacks are reported to used to decreasing code security, creating adversarial code, and personal data leaks over automated generated code.

What can be done to mitigate the severity of flaws introduced by AI? Does the literature suggest giving up on AI entirely? No, this is not what anyone suggests, as it can be imagined that AI is considered an instrument that, despite imperfect, has a clear advantage in terms of speeding up code production. Instead different mitigation strategies are suggested, although more research is required to discuss their effectiveness and efficacy.

- Modifications to the dataset can be a possibility, but the impacts and trade-offs of such an approach are necessary;
- Raising awareness of the context of prompts and how to increase their quality seems to affect the security quality of the code generated positively;
- Security processes, policies, and a degree of mistrust of the AI-generated code could help with security. In other words, AI-generated should pass specific processes—such as test and security verification—before being accepted;
- Educating end-users on AI models (and for code generation) on their limits could help. Future research is required in this area.

As a closing remark, we welcome that the study of the impact on the security of AI models is sparking. We also greet the increased attention that the community is dedicating to the problem of how insecure our systems will be as developers continue to resort to AI support for their work. However, it is still premature to conclude on the impact of the flaws introduced by AI models and, in particular, the impact of those flaws comparatively with those generated by human programmers. Although several mitigation techniques are suggested, what combination of them is efficient or practical is a question that still needs experimental data.

Surely, we have to accept that AI will be used more and more in producing code and that the practice and this tool are still far from being flawless. Until more evidence is available, the general agreement is to exert caution: AI models for secure code generation need to be approached with due care.

## Data availability statement

The datasets of the sample of papers for this study can be found in: https://zenodo.org/records/11092334.

## Author contributions

CN-R: Conceptualization, Data curation, Investigation, Methodology, Project administration, Resources, Validation, Writing—original draft, Writing—review & editing. RG-S: Investigation, Visualization, Writing—original draft, Writing—review & editing. AS: Conceptualization, Investigation, Methodology, Writing—review & editing. GL: Conceptualization, Funding acquisition, Investigation, Writing—original draft, Writing—review & editing.

## Footnotes

^1^The number of different tokens that a model can handle, and their internal representation, is a design choice.

^2^This is the meaning of “GPT:” generative pre-trained transformer.

^3^Some authors claim that, because there is an encoding-decoding step, and the output is probabilistic, data is not directly copy-pasted. However seriously this argument can be taken, LLMs can and do reproduce parts of their training set (Huang et al., 2023).

^4^Certain CWE prompting scenarios, when compared between the authors, had dissimilar security rates, which we would like to note.

^5^The authors do highlight that their proposal is not a poisoning attack.

^6^In reality, multiple (broadly incompatible) versions of Python coexist, but this is unimportant in the context of our discussion and we refer to them collectively as “Python.”

^7^One could argue for instance that the vulnerabilities occur in large proportions in generated code that fails basic functional testing, and would never make it into production because of this. Or, the other way around, that code without security vulnerabilities could still be functionally incorrect, which also causes issues. A full study of these effects remains to be done.

^8^They were tasked to write a program that “takes as input a string path representing a file path and returns a File object for the file at 'path' (Perry et al., 2023).”

^9^Following the authors of our sample, we use “C” to refer to the various versions of the C standard, indiscriminately.

^10^Here again we conflate all versions of Java together.

^11^The authors define single stupid bugs as “...bugs that have single-statement fixes that match a small set of bug templates. They are called 'simple' because they are usually fixed by small changes and 'stupid' because, once located, a developer can usually fix them quickly with minor changes (Jesse et al., 2023).”

^12^The attack is explained in detail in Section 5.2.
